# Fibroblast Activation Protein Specific Optical Imaging in Non-Small Cell Lung Cancer

**DOI:** 10.3389/fonc.2022.834350

**Published:** 2022-03-10

**Authors:** Layla Mathieson, Richard A. O’Connor, Hazel Stewart, Paige Shaw, Kevin Dhaliwal, Gareth O. S. Williams, Alicia Megia-Fernandez, Ahsan R. Akram

**Affiliations:** ^1^ Centre for Inflammation Research, Queen’s Medical Research Institute, University of Edinburgh, Edinburgh, United Kingdom; ^2^ Translational Healthcare Technologies Group, Centre for Inflammation Research, Queen’s Medical Research Institute, University of Edinburgh, Edinburgh, United Kingdom; ^3^ EaStCHEM, The University of Edinburgh School of Chemistry, Edinburgh, United Kingdom; ^4^ Cancer Research UK Edinburgh Centre, Institute of Genetics and Cancer, The University of Edinburgh, Edinburgh, United Kingdom

**Keywords:** optical, non-small cell lung carcinoma, imaging, fibroblast, FAP

## Abstract

Fibroblast activation protein (FAP) is a cell surface propyl-specific serine protease involved in the regulation of extracellular matrix. Whilst expressed at low levels in healthy tissue, upregulation of FAP on fibroblasts can be found in several solid organ malignancies, including non-small cell lung cancer, and chronic inflammatory conditions such as pulmonary fibrosis and rheumatoid arthritis. Their full role remains unclear, but FAP expressing cancer associated fibroblasts (CAFs) have been found to relate to a poor prognosis with worse survival rates in breast, colorectal, pancreatic, and non-small cell lung cancer (NSCLC). Optical imaging using a FAP specific chemical probe, when combined with clinically compatible imaging systems, can provide a readout of FAP activity which could allow disease monitoring, prognostication and potentially stratify therapy. However, to derive a specific signal for FAP any sequence must retain specificity over closely related endopeptidases, such as prolyl endopeptidase (PREP), and be resistant to degradation in areas of active inflammation. We describe the iterative development of a FAP optical reporter sequence which retains FAP specificity, confers resistance to degradation in the presence of activated neutrophil proteases and demonstrates clinical tractability ex vivo in NSCLC samples with an imaging platform.

## Introduction

Fibroblast activation protein-α (FAP) is a type II transmembrane glycoprotein which is a member of the serine protease family (1). FAP is minimally expressed by fibroblasts in health, but is highly expressed by activated fibroblasts which can be found in the stroma of epithelial tumours (2, 3). Furthermore, there is increasing evidence of the role of FAP in additional fibroproliferative conditions such as idiopathic pulmonary fibrosis, hepatic fibrosis, rheumatoid arthritis and myocardial infarction (4–7). Within tumours, FAP promotes tumour growth by promoting angiogenesis and ECM remodelling (8) and facilitates the progression of tumours by supressing the anti-cancer immune response (9, 10). High FAP expression is associated with poor survival, high recurrence rates and more advanced stage in several cancers, including oral squamous cell carcinoma, ovarian cancer, pancreatic ductal adenocarcinoma and non-small cell lung cancer (NSCLC) (11–15). Specifically in NSCLC, FAP expression has been associated with a higher peripheral neutrophil and lymphocyte count ratio and worse overall survival (16).

FAP has both endopeptidase activity (cleaving post proline peptide bonds of non-terminal amino acids) and exopeptidase activity (cleaving peptide bonds of terminal amino acids) (17) and is upregulated *in vitro* by TGF-β and IL-1β (18). Closely related peptidases include the dipeptidyl peptidases (DPPs), which have exopeptidase activity, and prolyl endopeptidase [PREP or prolyl oligopeptidase (POP)], which has endopeptidase activity (19). DPP-IV is highly expressed in many tissues (20), and PREP is a closely related peptidase which in the past has been used interchangeably with FAP (21, 22). As PREP has also been found to be present in the membrane of fibroblasts and is distinctive over FAP, selectivity is crucial (23).

Prior studies investigating FAP as an optical probe target focussed on near infrared (NIR) fluorophores where signal can be detected with minimal intrinsic tissue autofluorescence. Li et al. designed an activatable NIR fluorescent probe (ANP_FAP_) for FAP which was composed of the NIR dye Cy5.5 and the quencher dye QSY21 which were linked by a peptide sequence which is cleaved specifically by FAP (KGPGPNQC) (24). In murine tumour models the probe had a higher signal in FAP expressing tumours, but the study did not demonstrate selectivity over PREP. Although not developed as an imaging optical agent for *in vivo* use, Bainbridge et al. describe a FAP specific sequence for assaying circulating FAP, demonstrating a sequence with specificity over PREP (25).

Fluorescent probes are now being used in humans for detection of enzymatic activity in combination with technologies such as optical endomicroscopy (26, 27), and FAP presents an attractive target as there is significant upregulation in NSCLC (28). Optical imaging confers the advantages of a non-ionising source with high resolution imaging with dynamic readouts. When combined with endomicroscopy, this dynamic imaging method could allow for local monitoring of the FAP activity in patients through therapy.

This work aims to develop a FAP specific probe for use in humans with NSCLC that is specific over PREP and DPP-IV, resistant to degradation in areas of active inflammation and compatible with novel endomicroscopy platforms. We demonstrate development of a FAP specific probe that allows for both detection of fluorescence changes and fluorescence lifetime changes in NSCLC ex vivo, when used with an optical endomicroscopy platform.

## Materials and Methods

### Ethics Statement

Healthy volunteer blood was obtained following informed consent and the study was approved by Lothian Regional Ethics Committee (REC) (REC No: 20-HV-069) prior to enrolment in the studies. Cancer tissue was obtained following approval by NHS Lothian REC and facilitated by NHS Lothian SAHSC Bioresource (REC No: 15/ES/0094). All participants provided written informed consent. NSCLC tissues lung samples were collected from patients undergoing surgical resection with curative intent.

### Chemical Synthesis

Details of the chemical synthesis are provided in the **Supplementary Methods**.

### Probe Reconstitution

FAP1_Li-FAM_ and FAP1D_Li-FAM_ were reconstituted in DMSO to a stock solution of 10 mM and aliquots stored at -20°C. Fresh aliquots were reconstituted in the required buffer for each experiment. All other probes were water soluble and were reconstituted to 1 mM in deionised water and stocks frozen at -20°C.

### Recombinant Enzyme Reconstitution

Recombinant human enzymes [rhFAP (R&D Systems), rhPREP (R&D Systems) and rhDPPIV (Biolegend)] were stored in stocks at -70°C and diluted as required to be used at final concentration of 0.1 µg/ml. Enzymes were aliquoted upon receipt from the supplier and new stocks made from a fresh aliquot as required. Stocks were made at [4x] and control substrates of Z-Gly-Pro-AMC and H-Gly-Pro-AMC (Bachem) were used to confirm activity for each new stock of enzyme.

### Assessment With Recombinant Enzymes

Assays were undertaken in triplicate in blackened 384 well plates on ice prior to spectral reads. All solutions were diluted in TRIS buffer (25 nM Tris, 250 nM NaCl, pH 7.5). Probes were used at 5 µM and inhibitors (namely Talabostat (also referred to as Val-boroPro), Merck) at 10 µM, unless otherwise stated. All wells contained a final volume of 20 µl to include buffers, inhibitors (if used), substrates and recombinant human enzymes (at a concentration of 0.1 µg/ml unless otherwise stated). This equates to molarity concentrations of 1.16 micromolar for FAP, 1.23 micromolar for PREP and 1.16 micromolar for DPP-IV. Plates were sealed with an optical plate sealer (Biolegend) and were read in a preheated spectrophotometer (Synergy Biotek) in monochromator-based mode at 380/460 nm for control substrates and 480/530 nm for synthesised FAP probes.

### Assessment on Neutrophil Lysate

Neutrophils were isolated from peripheral blood of healthy volunteers by discontinuous percoll gradients, as previously described (29). Cells were resuspended in PBS (at 10 million cells/ml) and stimulated with 1 µM calcium ionophore, then lysed with 1% Triton-X-100. Lysate was used at 1:1 dilution for experiments, replacing recombinant enzymes in the assay as described above, with PBS as the buffer.

### Assessment on Cancer Associated Fibroblasts (CAFs)

CAFs were isolated from NSCLC patient samples as previously described (28). Briefly, tissue samples were minced with forceps and incubated for an hour in prewarmed RPMI media (Gibco) containing collagenase IV [2 mg/ml] (Sigma) and DNase [0.2 mg/ml] (Sigma). Samples were spun at 350 g for 5 minutes and red blood cells were lysed from samples using RBC lysis buffer (BioLegend) in 10 ml for 10 minutes. Following a further spin at 350 g for 5 minutes, cells were seeded in culture flasks in DMEM containing 10% FCS, 1% Penicillin-Streptomycin, 1% L-Glutamine and 10% Insulin Transferrin Selenium (ITS). 24 hours after seeding, non-adherent cells were washed from the flasks. Cells were maintained by standard cell culture methods and by passage 2, the predominant cell type was CAFs, assessed by flow cytometry markers and morphology. CAFs between passage 4-9 were used to assess FAP probes and confirmed to be FAP^hi^ by flow cytometry.

CAFs were seeded into 96 well plates in a 100 µl media (complete DMEM as described above) at a density of 1x10^5^ cells/ml to form a confluent monolayer within 24 hours. Wells containing cells had media removed and then were washed before buffer (DPBS) +/- inhibitors were added with the imaging probes. Immediately after adding imaging probes, the plate was transferred to the prewarmed spectrophotometer as above and read at 480/530 nm for one hour.

For imaging, CAFs were seeded in glass bottom chambers (Ibidi), grown, and fixed with 4% paraformaldehyde for 20 minutes at 4°C. Following three washes, cells were permeabilised with 0.2% Triton X-100, quenched with ammonia chloride (50mM) for 5 minutes and blocked with 1% BSA. FAP antibody at 1:100 (AF3715, R&D systems) was incubated overnight at 4°C, washed, incubated with secondary antibody (goat anti-sheep) conjugated to Alexa Fluor 633 (1:1000) and Alexa Fluor 488 Phalloidin 5 µL/250 µL (A12379, Life Technologies) for 1 hour and finally incubated with DAPI (D1306, Life Technologies) in the dark for 5 minutes. Images were taken on Leica SP5 confocal microscope (Leica Microsystems, Wetzlar, Germany) using dedicated laser excitation at 405 nm, 488 nm and 633 nm.

### Flow Cytometry

Cells collected in suspension (CAFs or neutrophils) were stained with a live/dead marker Zombie UV (1:1000, Biolegend) for 30 min at room temperature in DPBS (Gibco). Cells were then washed and stained with an anti-FAP-APC antibody (1:20, R&D Systems) for 20 mins at 4°C in DPBS supplemented with 2% FCS. After washing cells were fixed in a 1:1 solution of fixation buffer (Biolegend) and DPBS with 2% FCS overnight at 4°C before data acquisition on a LSR6Fortessa analyser (BD Biosciences). Flow cytometry data was then analysed using FlowJo version 10.7.1. Compensation was carried out using single stain control UltraComp eBeads (Invitrogen) and isotype control samples were stained using iso-anti-FAP-APC (1:20, R&D Systems). FAP expression was determined by gating on singlet, live cells and then looking at anti-FAP-APC signal compared to the isotype control.

### Fluorescence and Lifetime Imaging

For imaging solutions, varying concentrations of rhFAP were prepared in blackened eppendorfs and FAP3 to a final concentration of 5µM added and imaged as below. For biological specimens, NSCLC patient tissue samples from surgical resection were used fresh or snap frozen and stored at -80°C until required. Small fragments (approx. 4mm^3^) incubated at 37°C in a 96 well plate in phenyl-red free DMEM (Gibco) containing 10% FCS, 1% Penicillin-streptomycin and 1% L-Glutamine. For the tissue fragments to be used as inhibitor controls, we added Talabostat at a dilution of 1:1000. Samples were then imaged using a clinically approved fluorescence lifetime imaging system used in conjunction with an imaging fibre (30) providing a 400 x 400 µm field of view. The imaging system incorporates a pulsed supercontinuum laser source, in this case tuned for excitation at 488 nm, with an achromatic confocal laser scanning system and a time-resolved spectrometer. This spectrometer contains a 512 channel single photon avalanche diode (SPAD) sensor (31) allowing for the rapid collection of time resolved spectral fluorescence lifetime data. Fluorescence intensity and lifetime images were collected using an image resolution of 160 x 160 pixels over a 498 – 570 nm spectral range with an exposure time of 13 µs per pixel. This led to an imaging rate of ~3 frames per second.

For each condition, the tumour samples were imaged using a fibre placed at the surface of the tissue (baseline), and then again at 10-minute intervals following the additions of equimolar concentrations of probe +/- Inhibitor. For imaging solutions, the fibre was held within the solution and imaged every 5 minutes. Each condition and time-point data for both fluorescence intensity and lifetime were collected. Imaging sequences had non-relevant frames removed, and the entire field of view was analysed. Analysis was undertaken using a bespoke software suite utilising the rapid lifetime determination (RLD) method (32). The RLD method utilises two-time bins for reduced data load and high-speed analysis whilst retaining reasonable lifetime approximation for single exponential decays. Whilst the sample analyses are likely multi-exponential in character the lightweight RLD algorithm provides a good approximation to the intensity weighted average lifetime observed, providing sufficient discrimination of sample type. Data had background subtraction, lightfield normalisation and an intensity threshold (of 20 counts) applied through all sequences. Intensity data are provided as relative units (RU) and lifetime as nanoseconds (ns).

## Results and Discussion

### Optimizing Fragment for FAP Specificity

**Figure 1:**
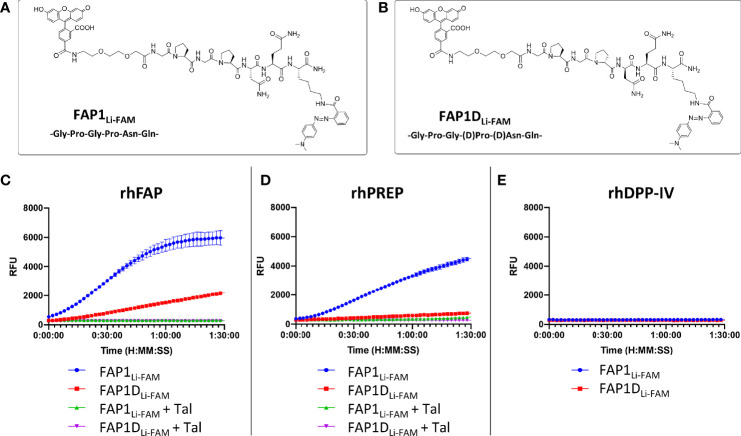
FAP1 probes with recombinant human enzymes rhFAP, rhPREP and rhDPPIV to assess endo and exopeptidase activity. **(A)** The structure of FAP1_Li-FAM_; **(B)** The structure of FAP1D_Li-FAM_; **(C)** Each probe (at 5µM) incubated with rhFAP with and without the inhibitor Talabostat (Tal). FAP1_Li-FAM_ is cleaved rapidly by rhFAP, FAP1D_Li-FAM_ shows lower rate of cleavage and Talabostat inhibits both probes; **(D)** Probes (5µM) with rhPREP demonstrating cleavage of FAP1_Li-FAM_ and FAP1D_Li-FAM_, although FAP1_Li-FAM_ is cleaved more rapidly. Talabostat inhibits cleavage of both probes by rhPREP; **(E)** Probes (5µM) with DPP-IV, demonstrating no activity. Representative plots shown, n=3 for each experiment, run in triplicate.

As starting point for our study, a peptide sequence previously published within a FAP optical reporter was synthesised (GPGPNQ), which had been determined to be FAP specific over DPP-IV but had not been assessed against PREP (24). We synthesised the reported peptide sequence but for FRET pairing we utilised a Carboxyfluorescein (FAM) fluorophore with a Methyl Red quencher (**Figure 1A**) as an alternative to Cy5.5/QSY21. The peptide sequence was synthesized by Fmoc solid-phase peptide synthesis on ChemMatrix resin using Oxyma/DIC as the coupling combination. FAM was incorporated at the N-terminal of the peptide after an ethylenglycol unit, and Methyl Red was added at the side chain of a Lys residue at the C-terminal. All probes were purified and characterized by RP-HPLC and MALDI TOF MS (ESI for details). Assessment of this probe (termed FAP1_Li-FAM_) sequence against the recombinant enzymes FAP and PREP demonstrated endopeptidase activity through PREP as well as FAP (**Figures 1C, D**), which was independently demonstrated by Bainbridge et al. (25) for the same sequence. Confirming previous reports, DPP-IV did not cleave the probe (**Figure 1E**) signifying absent exopeptidase activity as the proline residues are not in terminal positions. For inhibition we used Talabostat, also known as Val-boroPro, which is a non-selective inhibitor of dipeptidyl peptidases (DPPs), including DPP-IV, DPP-8, DPP-9, fibroblast activation protein (FAP), and prolyl endopeptidase. The IC_50_ (nmol/l) for DPP-IV, FAP and PREP are 4, 390 and 560 respectively (33). Talabostat successfully abrogated the signal (**Figure 1D**).

The construct has Gly-Pro repeats susceptible to PREP cleavage, therefore modifications to block the endopeptidase action by the second proline were made with D-proline and D-asparagine (GPGpnQ) to try and confer a uniquely FAP cleavable probe (FAP1D_Li-FAM_ structure shown in **Figure 1B**). This demonstrated partial reduction in activity in the presence of PREP (**Figure 1D**), but also demonstrated that FAP activity was significantly reduced (**Figure 1C**). Further iterations were assessed against FAP1_Li-FAM_ and FAP1D_Li-FAM_ activity to assess cleavage efficacy.

**Figure 2:**
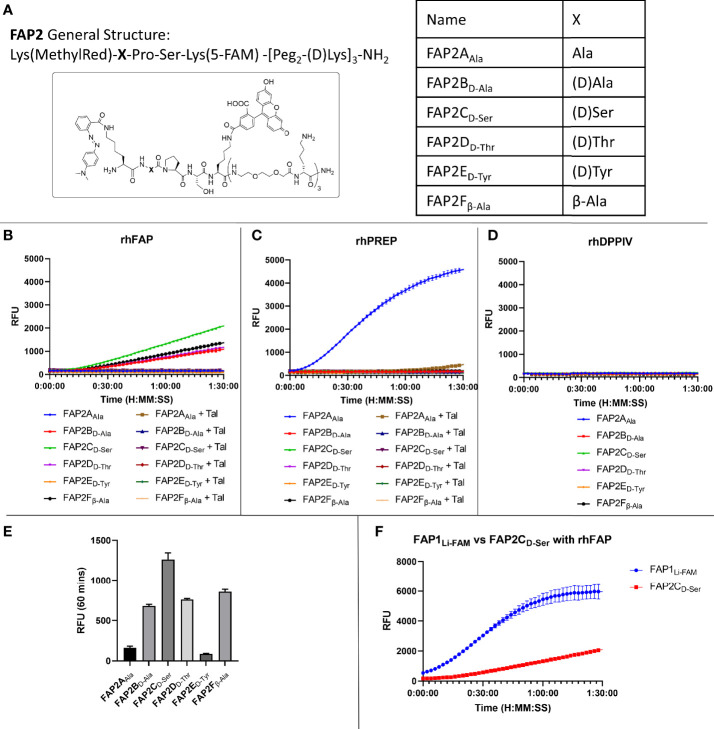
Iterations of probe FAP2 to determine a sequence specific for FAP over PREP. **(A)** The structure of FAP2 probes and the different variations in the amino acid X; **(B)** Assessment of the probes (5µM) against rhFAP showing relative intensity and Talabostat (Tal) inhibiting cleavage. FAP2C_D-Ser_ shows highest rate of cleavage; **(C)** Assessment of the probes (5µM) against rhPREP showing iteration FAP2A_Ala_ is cleaved by rhPREP; **(D)** Assessment of the probes against DPP-IV (5µM) showing none of the iterations are cleaved; **(E)** Comparison of the relative fluorescence intensity of each probe iteration at 60 minutes; **(F)** Comparison of FAP1_Li-FAM_ with FAP2C_D-Ser_ showing the signal intensity of FAP1 is higher within 90 minutes. Representative images, n=3, mean RFU plotted in bar graph and error bars show standard deviation.

Whilst there was an improvement in the specificity, FAP1D_Li-FAM_ still demonstrated PREP cleavage so additional compounds were synthesised to overcome this. Shorter versions with modified sequences were synthesised, and the amino acid prior to proline altered in six iterations (structures shown in **Figure 2A**), as it had been previously reported that such modification can introduce FAP specificity (25). At the carboxy-terminus of the peptide, two replicates of bisethyleneglycol and D-lysine were added to ensure both solubility and stability against proteases (34). These six different compounds were then reassessed with recombinant human enzymes (**Figures 2B–D**). Insertion of Ala (FAP2A_Ala_) demonstrated cleavage by PREP and FAP activity was absent, and the insertion of D-Tyr also resulted in loss of FAP activity. The iterations containing D-Ala, D-Ser, D-Thr and β-Ala in the position prior to proline all demonstrated FAP specificity over PREP, however, with reduced signal that may preclude clinical translation. Assessment against DPP-IV confirmed no iterations had introduced exopeptidase activity (**Figure 2D**). As the clinical setting requires rapid optical readout, two further iterations containing D-Ser (FAP3 and FAP-sp) were made inspired by FAP2C_D-Ser_ results, which demonstrates FAP specificity (**Figures 2B, C**) without PREP cleavage whilst displaying the strongest signal compared to the other iterations (**Figure 2E**). Comparison with the FAP1_Li-FAM_ compound demonstrated a low signal (**Figure 2F**), and this served as a comparison to additional compounds.

### Improvement of Signal-to-Noise

**Figure 3:**
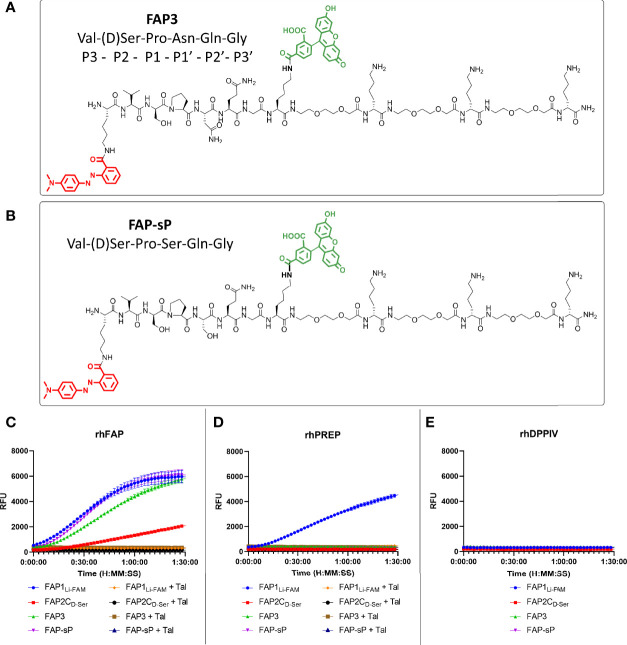
FAP3 assessment against FAP-sP demonstrates FAP specificity. **(A)** The structure of FAP3; **(B)** The structure of FAP-sp; **(C)** Assessment of FAP3 and FAP-sP (all 5µM) when compared to prior iterations.; **(D)** rhPREP assessment demonstrating no rhPREP cleavage for FAP3 or FAP-sP; **(E)** DPPIV assessment demonstrating no exopeptidase activity. Representative plots, n=3 for each experiment run in triplicate.

Using the D-Ser iteration we increased the peptide chain to include Asn to derive a novel sequence (**FAP3**: Lys(MethylRed)-Val-(D)Ser-Pro-Asn-Gln-Gly-Lys(5-FAM)-[Peg_2_-(D)Lys]_3_-NH_2_) or with Ser (**FAP-sP**) and to act as a comparator (structures shown in **Figures 3A, B**). FAP-sP was previously reported by Bainbridge to be utilised as a serum FAP detection probe (25). Both sequences demonstrated FAP specificity (**Figure 3C**) with improved signal characteristics which were further confirmed by MALDI-TOF analysis (**Supplementary Figure S1**). Testing also confirmed no cleavage by PREP or DPP-IV for either compound (**Figures 3D, E**).

### Assessment of Probe in Biological Environments

**Figure 4:**
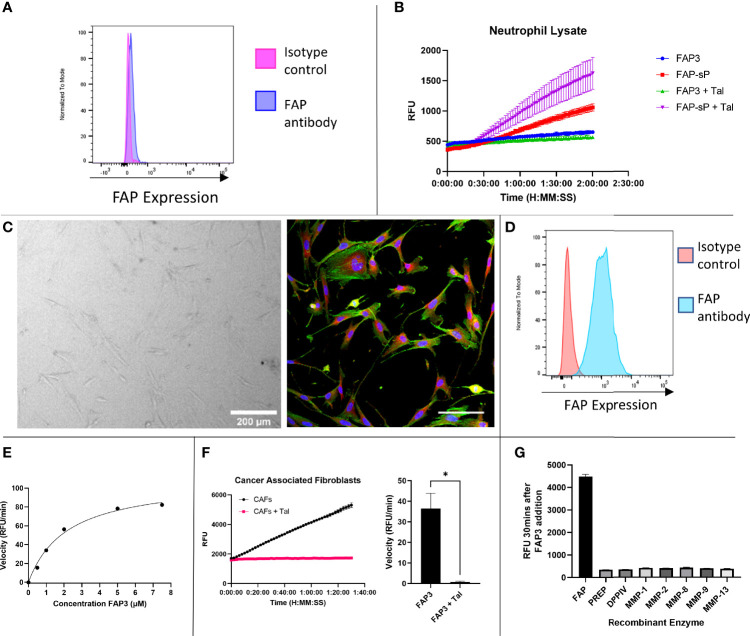
FAP3 and FAP-sP probes assessed on activated neutrophil lysate and cancer associated fibroblasts (CAFs). **(A)** Flow cytometry confirmed that neutrophils are FAP negative; **(B)** Assessment of FAP3 and FAP-sP in activated neutrophil lysate demonstrated cleavage of FAP-sP, including in the presence of Talabostat (Tal), but no cleavage of FAP3; **(C)** Cancer associated fibroblasts in culture (brightfield, 200µm scale bar) and antibody stained for FAP (red), DAPI (blue) and phalloidin (green), scale bar 100µm; **(D)** Flow cytometry confirmed CAFs are FAP positive; **(E)** Enzyme kinetics of using varying concentrations of substrate FAP3 in presence of rhFAP; **(F)** When incubated with CAFs FAP3 (5µm) was cleaved and signal was abrogated in the presence of Talabostat; representative plot on left and sum of n=3 with calculation of velocity in RFU/min shown on right.; **(G)** FAP3 demonstrates no activity for other recombinant enzymes (MMPs) likely to be present in NSCLC. n=2 for neutrophil lysate testing and MMP testing, n=3 for CAF testing, each experiment run in triplicate. Representative images shown *p < 0.05.

To assess whether the sequence had sufficient robustness for clinical translation we assessed whether; i) the probe remains intact in areas of low FAP but high protease activity, and ii) the imaging probe can detect physiological levels of FAP. To ensure specificity is maintained in an inflamed environment the probe was assessed against activated neutrophils as these are one of the most predominant leucocyte cell subtypes in cancer (35). Neutrophils were confirmed to be negative for FAP (**Figure 4A**, gating strategy **Supplementary Figure S2**). **FAP3** was stable in the presence of neutrophil lysate, however, **FAP-sP** was cleaved (**Figure 4B**) in a non-FAP dependent manner. Subsequent analysis by MALDI-TOF revealed all **FAP-sP** probe was cleaved by activated neutrophil lysate, but **FAP3** remained intact (**Supplementary Figures S3A, B**). The mechanism causing cleavage/degradation of FAP-sP remains unclear and may include additional proteases from activated neutrophils acting on the substrate, however this provides additional confidence for FAP3 as it remains intact.

Cancer associated fibroblasts were isolated and cultured from NSCLC patient samples (**Figure 4C**) and confirmed to be FAP expressing (**Figure 4D**, gating strategy **Supplementary Figure S4**), in line with our previous work (28). FAP3 was incubated with increasing concentrations of substrate (**Figure 4E**), which demonstrated a K_M_ value of 2.168 (SE +/- 0.3736) µM and V_max_ of 109.5 (SE +/-7.119) RFU/min. For **FAP3** this equates to a k_cat_ of 0.081236 (+/-0.005281) min^-1^ and a k_cat_/K_M_ ratio of 3.747x10^4^ M^-1^min^-1^. **FAP3** was cleaved by FAP^+^CAFs, with inhibition of signal when co-incubated with Talabostat (**Figure 4F**). Finally, to ensure stability in the presence of other matrix remodelling proteases found to be upregulated in NSCLC, FAP3 was assessed against a panel of MMP’s including MMP 2,9,13 demonstrating no activity (**Figure 4G**). Therefore, FAP3 is a FAP specific sequence over PREP, DPP-IV and MMP’s, that can detect FAP within physiological levels and remains resistant to non-specific inflammatory cell degradation.

### Fluorescence Lifetime Imaging (FLIM) of NSCLC Tissue

Label free optical imaging modalities have the potential to characterize lung cancer using both optical endomicroscopy and fluorescence lifetime imaging (FLIM) systems (36). Here we used a clinically approved fluorescence and FLIM imaging system (31) which was compatible with bronchoscopy, where a fibre can be passed through the working channel to access tumours and lung parenchyma (**Supplementary Figure S5**). FLIM imaging utilizes the exponential decay rate of the photon emission from the fluorophores (fluorescence lifetime) to create the image, which provides additional data over fluorescence intensity alone. This system is entering clinical trials and can simultaneously measure fluorescence intensity and lifetime based imagery.

To assess the ability of the system to detect both fluorescence intensity and lifetime changes over time, varying concentrations of rhFAP were incubated with 5µM FAP3 and imaged for up to 40 minutes (**Supplementary Figure S6**). Increases in both fluorescence intensity and lifetime were demonstrated, within 5 minutes, over time showing the capability of the FLIM imaging modality to track changes in these parameters in relevant concentrations and timeframes. As the concentration of rhFAP increased, a corresponding increase in both fluorescence intensity and lifetime was observed.

**Figure 5:**
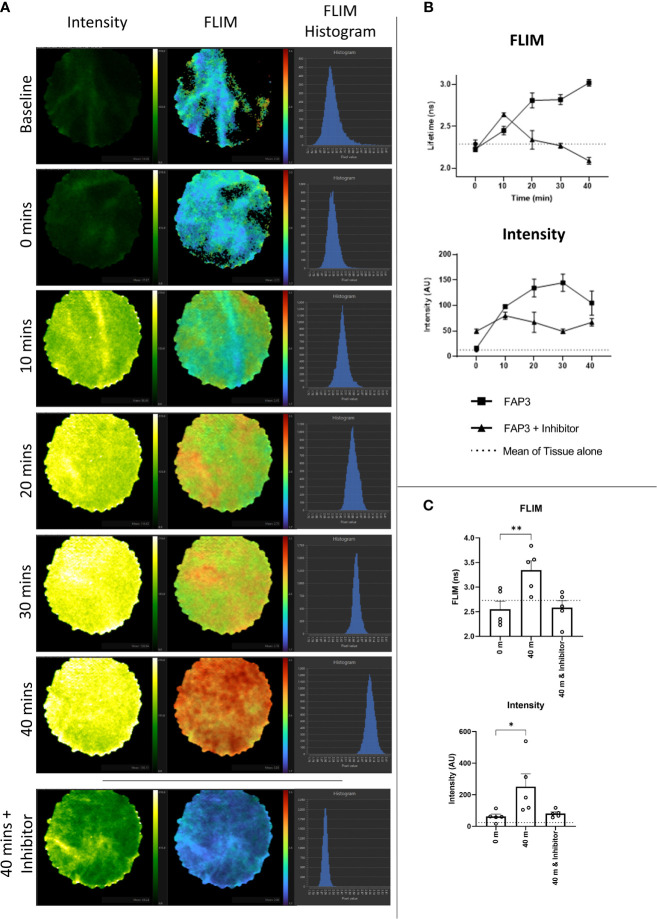
Fluorescence lifetime imaging in NSCLC tissue using FAP3 probe and a clinically approved FLIM system. **(A)** Representative images showing the change in fluorescence intensity and lifetime after 488nm excitation of a NSCLC tumor tissue sample over time when incubated with FAP3 at 5µM, with lowest panel demonstrating the late time point with presence of the inhibitor Talabostat; **(B)** Representative plots showing the change in intensity and fluorescence lifetime over 40 mins on a NSCLC tissue sample; **(C)** Aggregate analysis across 5 NSCLC samples at 40 minutes demonstrating a significant increase in fluorescence lifetime and intensity, with inhibition in the presence of Talabostat. N=5, analysis by paired t-test, *p < 0.05, **p < 0.01.

Next, to assess if using FAP3 we could detect FAP specific cleavage (measured by a change in fluorescence intensity or lifetime) in biological samples we utilized ex vivo lung cancer specimens including two adenocarcinomas, two squamous cell carcinoma and one adenosquamous carcinoma. Representative images showing the intensity and FLIM measurements taken from the imaging system are shown in **Figure 5A** and the change in FLIM and intensity over time for this sample are shown in **Figure 5B**, further showing the increasing signals with time, related to the presence of FAP. All tumours demonstrated a baseline intrinsic autofluorescence signature that was used to track relative changes against the presence of probes (**Supplementary Figure S7**). Across all samples there was an increase in fluorescence intensity and a change to a longer lifetime signature following the addition of FAP3 over time, both of which were abrogated by the inhibitor Talabostat. Summation of the data demonstrated significant FAP dependent increase in both intensity and longer lifetime across all samples (**Figure 5C**). Assessing the data on a per cancer basis (**Supplementary Figure S7**) there are two interesting features to note - the FLIM signature of the intact probe becomes the dominant signature irrespective of the intrinsic autofluorescence signature and secondly, the rate of change varies amongst the different samples (CR68 and CR126 demonstrate maximum change within 10 minutes). This was most apparent for CR126 where the addition of the probe resulted in demonstration of immediate changes in FLIM ahead of the detectable fluorescence intensity increase for the same sample (**Supplementary Video 1**). Together, this demonstrates FAP3 can detect FAP specific activity in NSCLC using both changes in fluorescence intensity and lifetime and the higher rate of cleavage indicates presence of a higher concentration of FAP. The tracking of these dynamics was made possible by the high acquisition speed of the imaging system.

## Conclusions

We have developed an optical imaging probe capable of FAP imaging within physiological levels in NSCLC patient derived cancer associated fibroblasts and demonstrated changes in fluorescence intensity and lifetime in several patient samples using an imaging system undergoing clinical translation. Furthermore, we have demonstrated specificity over PREP, a closely related endopeptidase to FAP, DPP-IV and MMPs and demonstrated stability in highly proteolytic conditions by testing against activated neutrophils. We have therefore developed a FAP specific optical imaging probe which has potential applications in imaging of NSCLC as well as other FAP mediated inflammatory conditions.

## Data Availability Statement

The original contributions presented in the study are included in the article/**Supplementary Material**. Further inquiries can be directed to the corresponding author.

## Ethics Statement

The studies involving human participants were reviewed and approved by Lothian Regional Ethics Committee (REC) (REC No: 20-HV-069) and NHS Lothian REC and facilitated by NHS Lothian SAHSC Bioresource (REC No: 15/ES/0094). The patients/participants provided their written informed consent to participate in this study.

## Author Contributions

LM undertook *in vitro* and ex vivo experimentation. RO’C undertook supervision and *in vitro* and ex vivo experimentation. PS undertook MALDI analysis. AM-F designed and synthesized the probes. HS, KD, and GW developed the FLIM imaging system and analysis methods and supported the clinical translational development of the imaging system and software. AA undertook conception, design of work and supervised the project as well as experimentation. LM and AA wrote the manuscript. All authors approved the manuscript.

## Funding

This work was funded by a Cancer Research UK Clinician Scientist Fellowship award to ARA (A24867). LM and PS are funded through the EPSRC and MRC Centre for Doctoral Training in Optical Medical Imaging (EP/L016559/1). AM-F acknowledges Engineering and Physical Sciences Research Council (EPSRC, United Kingdom, grant number EP/R005257/1).

## Conflict of Interest

LM, AMF and ARA are named on a patent relating to the probe construct, filed by The University of Edinburgh. GOSW is named on a patent filed by The University of Edinburgh related to the imaging system.

The remaining authors declare that the research was conducted in the absence of any commercial or financial relationships that could be construed as a potential conflict of interest.

## Publisher’s Note

All claims expressed in this article are solely those of the authors and do not necessarily represent those of their affiliated organizations, or those of the publisher, the editors and the reviewers. Any product that may be evaluated in this article, or claim that may be made by its manufacturer, is not guaranteed or endorsed by the publisher.
