# Supplementary material for: Fibroblast Activation Protein Specific Optical Imaging in Non-Small Cell Lung Cancer
Source: Front Oncol. 2022 Mar 10;12:834350. doi: 10.3389/fonc.2022.834350 (PMC8961646; doi:10.3389/fonc.2022.834350)
Supplement: Supplementary file 1 [file DataSheet_1.docx]

Supplementary Methods and Materials

Fibroblast Activation Protein specific optical imaging in Non-Small Cell Lung Cancer.

Layla Mathieson, Richard O’Connor, Hazel Stewart, Paige Shaw, Kevin Dhaliwal, Gareth O S Williams, Alicia Megia-Fernandez, Ahsan R Akram

Supplementary Methods

Supplementary Table

Supplementary Figures S1-7

**Supplementary Methods**

**Chemistry Synthesis Methods**

Commercially available reagents were used without further purification. Methyl Red (MR) was purchased from Sigma, 5-Carboxyfluorescein (5-FAM) was purchased from Carbosynth. Ltd., [2-[2-(Fmoc-amino)ethoxy]ethoxy]acetic acid was purchased from Iris Biotech. Analytical reverse-phase high-performance liquid chromatography (RP–HPLC) was performed on an Agilent 1100 system equipped with a Kinetex XB-C18 reverse-phase column (50 x 4.6 mm, 5 μm) with a flow rate of 1.0 mL/min and eluting with H_2_O/CH_3_CN/HCOOH (95/5/0.1) to H_2_O/CH_3_CN/HCOOH (5/95/0.1) over 6 min, holding at 95% CH_3_CN for 2 min, with detection at 254 nm and 495 nm and by evaporative light scattering. Semi-preparative RP–HPLC was performed on an Agilent 1100 system equipped with a Zorbax Eclipse XDB-C18 reverse-phase column (250 x 9.4 mm, 5 μm) with a flow rate of 2.0 mL/min and eluting with 0.1% HCOOH in H_2_O (A) and 0.1% HCOOH in CH_3_CN (B), with a gradient of 5 to 95% B over 30 min and additional isocratic period of 5 min. Electrospray ionization mass spectrometry (ESI–MS) analyses were carried out on an Agilent Technologies LC/MSD Series 1100 quadrupole mass spectrometer (QMS) in ESI mode. MALDI spectra were acquired on a Bruker Ultraflextreme MALDI-TOF MS with a matrix solution of sinapic acid (10 mg/mL) in H_2_O/CH_3_CN/TFA (50/50/0.1).

**General solid-phase synthesis methods**

Manual peptide synthesis was performed on aminomethyl-ChemMatrix resin using an Fmoc-protected Rink amide linker. General procedures were as follows:

**Coupling of Rink amide linker**

The Fmoc-Rink linker (4-[(R,S)-a-[1-(9H-Fluoren-9-yl)-methoxy-formamido]-2,4-dimethoxybenzyl-phenoxyacetic acid) (0.54 g, 1.0 eq) was dissolved in DMF (10 mL) and Oxyma (0.14 g, 1.0 eq.) was added and the mixture was stirred for 10 min. Diisopropylcarbodiimide (DIC, 155 µL, 1.0 eq.) was then added and the solution stirred for 1 min before adding it to aminomethyl-ChemMatrix resin (1.0 g, 1.0 mmol/g). The resulting mixture was stirred at 50⁰C for 45 min and washed with DMF (3x10 mL), DCM (3x10 mL) and MeOH (3x10 mL). Finally the resin was treated with Ac_2_O:Py:DMF (2:3:15) for 30 min in order to cap any remaining free amino groups and was washed again with DMF (3x10 mL), DCM (3x10 mL) and MeOH (3x10 mL). Resin loading was calculated as ~0.58 mmol/g via spectrophotometric test.

**Fmoc deprotection:** to the resin pre-swollen in DCM 20% piperidine in DMF was added and the mixture stirred for 2x10 min. The solution was drained and the resin washed with DMF (3x10 mL), DCM (3x10 mL) and MeOH (3x10 mL).

**Aminoacid coupling:** A solution of the appropriate D or L-amino acid (3.0 eq per amine) and Oxyma (3.0 eq) in DMF (0.1M) were stirred for 10 min. DIC (3.0 eq) was added and stirred for 1 min. The pre-activated mixture was then added to the resin pre-swollen in DCM and the reaction heated at 50⁰C for 30 min. The solution was drained and washed with DMF (3x10 mL), DCM (3x10 mL) and MeOH (3x10 mL). The completion of the coupling reactions was monitored by Kaiser or Chloranil tests (when secondary amines are involved). Fmoc-Lys(Dde)-OH was used as an orthogonal reagent to allow introduction of the dyes. Fmoc-Lys(Methyl Red)-OH was used as building block to add the quencher.

**Coupling of other carboxylic acids:** Coupling of {2-[2-(Fmoc-amino)ethoxy]ethoxy}acetic acid (PEG) and 5-Carboxyfluorescein (5-FAM) was carried out following the same procedure as described for the aminoacid couplings.

**Dde deprotection** in presence of Fmoc protecting group was achieved as previously reported [27] : 1.25 g of NH_2_OH^.^HCl and 0.918 g of imidazole were suspended in 5 mL of NMP and the mixture was sonicated until complete dissolution; 5 volumes of this solution were diluted with 1 volume of CH_2_Cl_2_ and added to the resin. After 3h the solution was drained and the resin washed with DMF (3x10 mL), DCM (3x10 mL) and MeOH (3x10 mL).

**Cleavage and purification:** The resin (100 mg) pre-swollen in DCM was treated with a cleavage cocktail of TFA:triisopropylsilane(TIS):water (95:2.5:2.5) (2 mL) for 3h. The reaction solution was drained and the resin washed with the cleavage cocktail (1 mL). The combined solution was precipitated against cold ether, centrifuged (x3) and purified by RP-HPLC on a C-18 semi-preparative column. The desired fractions containing the product were collected and lyophilized to afford the pure compounds.

**Characterization table**

| **Compound** | **Sequence** | **MW** (Da) | **MALDI**  **m/z_obs_**  (Da)^a^ | **HPLC**  **t_R_** (min)^b^ |
| --- | --- | --- | --- | --- |
| **FAP1_Li-FAM_** | (FAM)-PEG_2_-**GPGPNQ**-K(MR)-NH_2_ | 1450.53 | 1451.04 | 3.963 |
| **FAP1D_Li-FAM_** | (FAM)-PEG_2_-**GPGpnQ**-K(MR)-NH_2_ | 1450.53 | 1451.31 | 4.006 |
| **FAP2A_Ala_** | K(MR)-**APS**-K(FAM)-(PEG_2_-k)_3_-NH_2_ | 1958.25 | 1959.32 | 2.816 |
| **FAP2B_D-Ala_** | K(MR)-**aPS**-K(FAM)-(PEG_2_-k)_3_-NH_2_ | 1958.25 | 1959.72 | 3.068 |
| **FAP2C_D-Ser_** | K(MR)-**sPS**-K(FAM)-(PEG_2_-k)_3_-NH_2_ | 1974.25 | 1976.56 | 2.895 |
| **FAP2D_D-Thr_** | K(MR)-**tPS**-K(FAM)-(PEG_2_-k)_3_-NH_2_ | 1988.27 | 1989.42 | 2.995 |
| **FAP2E_D-Tyr_** | K(MR)-**yPS**-K(FAM)-(PEG_2_-k)_3_-NH_2_ | 2050.35 | 2051.51 | 3.078 |
| **FAP2F_β-Ala_** | K(MR)-**βAPS**-K(FAM)-(PEG_2_-k)_3_-NH_2_ | 1958.25 | 1959.42 | 2.836 |
| **FAP3** | K(MR)-**VsPNQG**-K(FAM)-(PEG_2_-k)_3_-NH_2_ | 2285.59 | 2287.39 | 2.805 |
| **FAP-sP** | K(MR)-**VsPSQG**-K(FAM)-(PEG_2_-k)_3_-NH_2_ | 2258.56 | 2260.38 | 2.824 |
|  |  |  |  |  |

^a^ m/z observed corresponding to [M+H]^+^; ^b^ t_R_ for elution with 0.1% HCOOH in H_2_O (A) and 0.1% HCOOH in CH_3_CN (B), with a gradient of 5 to 95% B over 6 min, holding at (B) for 2 min.

**MALDI Analysis of probes in biological assays**

MALDI spectra were acquired on a Bruker Ultraflextreme MALDI-TOF MS. Neutrophil lysate experiment samples were prepared by plating 1 µl of the sample with 1 µl of the matrix solution sinapic acid (10 mg/mL) in H_2_O/CH_3_CN/TFA (70/30/0.1). Recombinant enzyme experiment samples were prepared using 1 µl of the sample with 1 µl of the matrix solution α-cyano-4-hydroxycinnamic acid (10 mg/mL) in H_2_O/CH_3_CN/TFA (50/50/0.1)

**Calibration Curve**

A calibration curve was determined using the FAP3 concentration (C-terminal cleavage fragment) against RFU to calculate the enzyme kinetic data and is shown below.


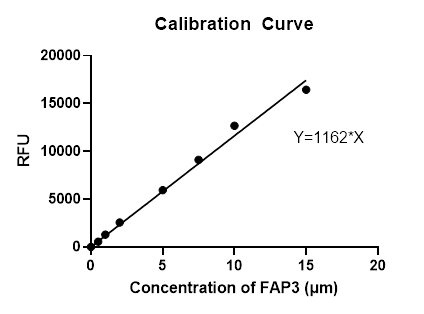

FAP2 Compounds

**Supplementary Table 1: Summary showing amino acid chain used for each probe**

| Probe | Sequence |
| --- | --- |
| FAP1_Li-FAM_ | FAM-Peg2-Gly-Pro-Gly-Pro-Asn-Gln-Lys(MethylRed)-NH2 |
| FAP1D_Li-FAM_ | FAM-Peg2-Gly-Pro-Gly-(D)Pro-(D)Asn-Gln-Lys(MethylRed)-NH2 |
| FAP2A_Ala_ | Lys(MethylRed)- Ala-Pro-Ser-Lys(5-FAM)-[Peg_2_-(D)Lys]_3_-NH_2_ |
| FAP2B_D-Ala_ | Lys(MethylRed)-(D)Ala-Pro-Ser-Lys(5-FAM)-[Peg_2_-(D)Lys]_3_-NH_2_ |
| FAP2C_D-Ser_ | Lys(MethylRed)-(D)Ser-Pro-Ser-Lys(5-FAM)-[Peg_2_-(D)Lys]_3_-NH_2_ |
| FAP2D_D-Thr_ | Lys(MethylRed)-(D)Thr-Pro-Ser-Lys(5-FAM)-[Peg_2_-(D)Lys]_3_-NH_2_ |
| FAP2E_D-Tyr_ | Lys(MethylRed)-D-Tyr-Pro-Ser-Lys(5-FAM)-[Peg_2_-(D)Lys]_3_-NH_2_ |
| FAP2F_β-Ala_ | Lys(MethylRed)-(β)Ala-Pro-Ser-Lys(5-FAM)-[Peg_2_-(D)Lys]_3_-NH_2_ |
| FAP3 | Lys(MethylRed)-Val-(D)Ser-Pro-Asn-Gln-Gly-Lys(5-FAM)-[Peg2-(D)Lys]3-NH2 |
| FAP-sP | Lys(MethylRed)-Val-(D)Ser-Pro-Ser-Gln-Gly-Lys(5-FAM)-[Peg2-(D)Lys]3-NH2 |

**Supplementary Figures
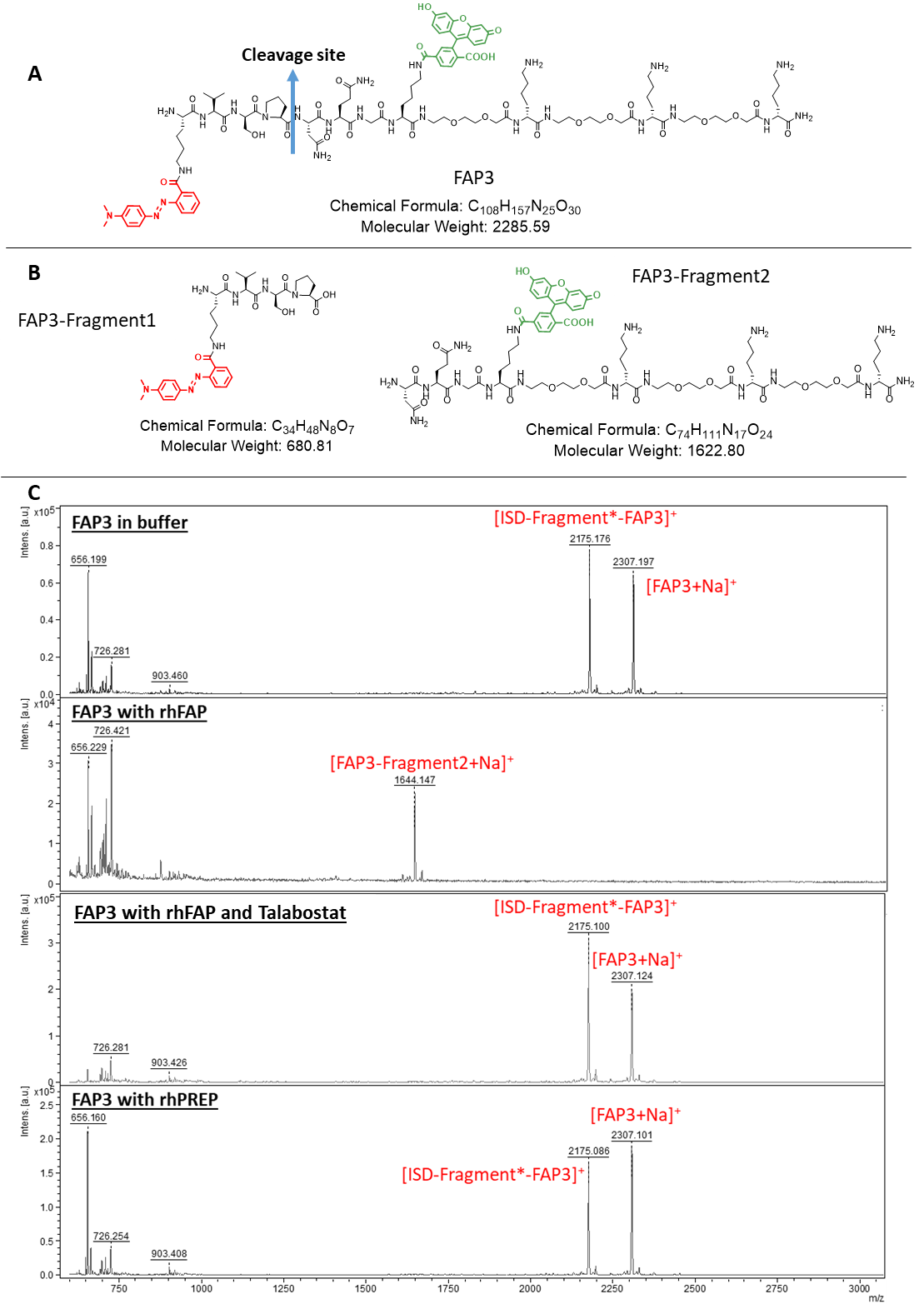
**

**Figure S1: The cleavage of FAP3 confirmed by MALDI**. (A) The complete structure of FAP3 showing the molecular weight and the site of cleavage post proline; (B) The fragments and their corresponding molecular weights expected when the probe is cleaved post proline; (C) MALDI analysis confirming the cleavage of FAP3 by rhFAP and no cleavage in the presence of rhFAP with Talabostat or rhPREP. *In-source decay (ISD) fragmentation of FAP3 that occurs in the MALDI source prior to ion extraction


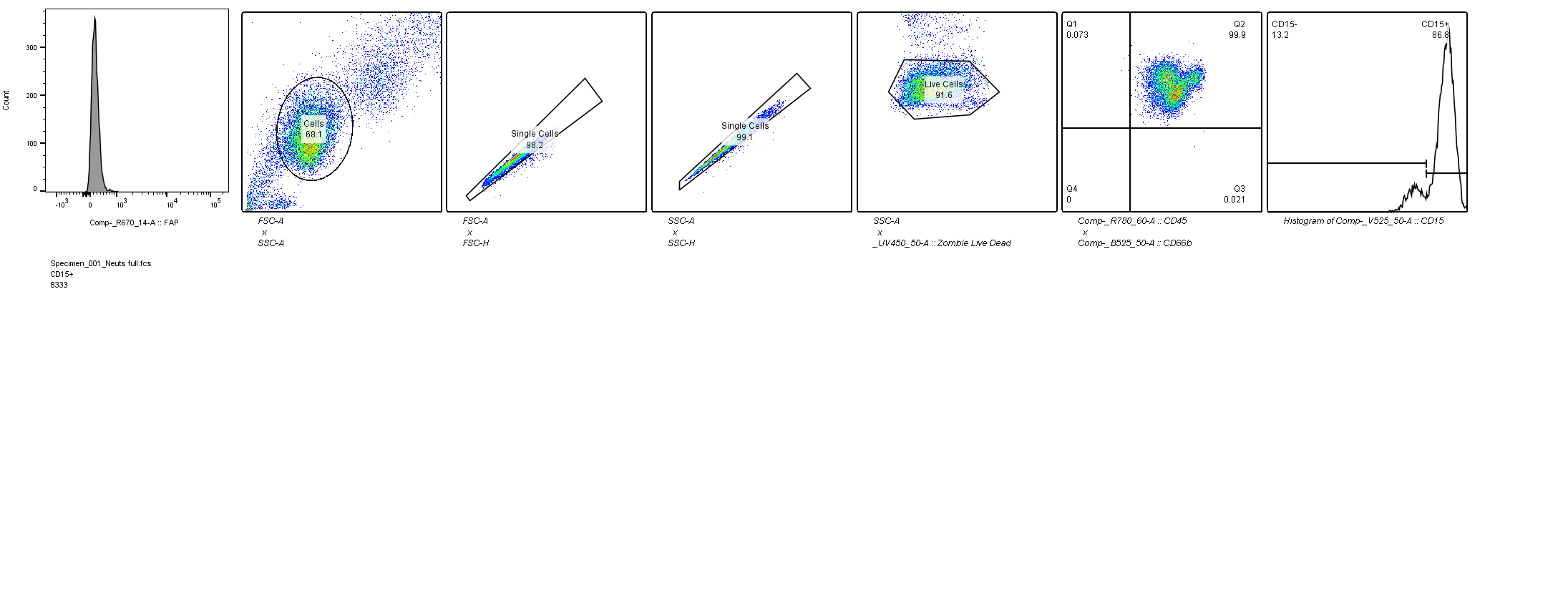


FAP Expression

Gate on cell population expected to be neutrophils due to prep method and size

Gate on single cells (doublet exclusion)

Select CD45+, CD66b+ cells

Confirm neutrophils by selecting CD15+ cells

Gate on live cells using viability dye staining


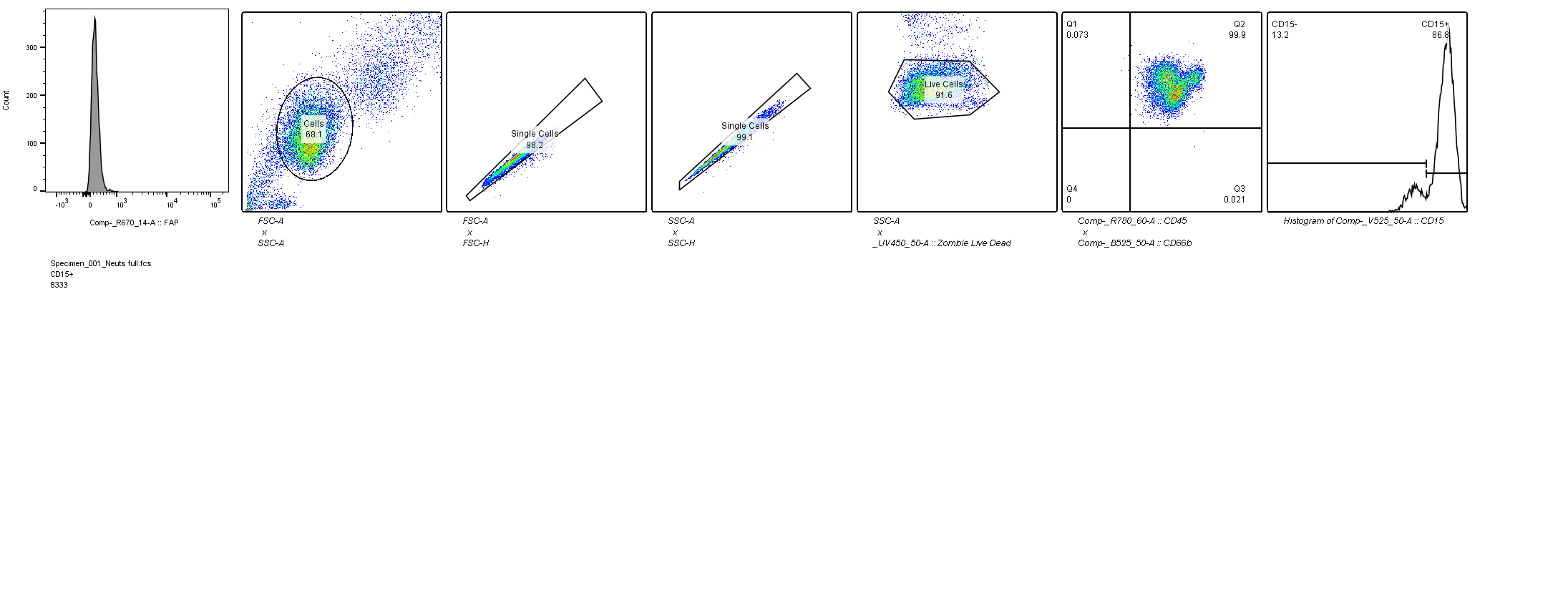


**Figure S2: Gating strategy used to confirm cells were neutrophils and measure FAP expression.** Gating strategy demonstrating sequential gating on cells, singlet cells, live cells and CD45^+^, CD66b^+^ and CD15^+^ cells.


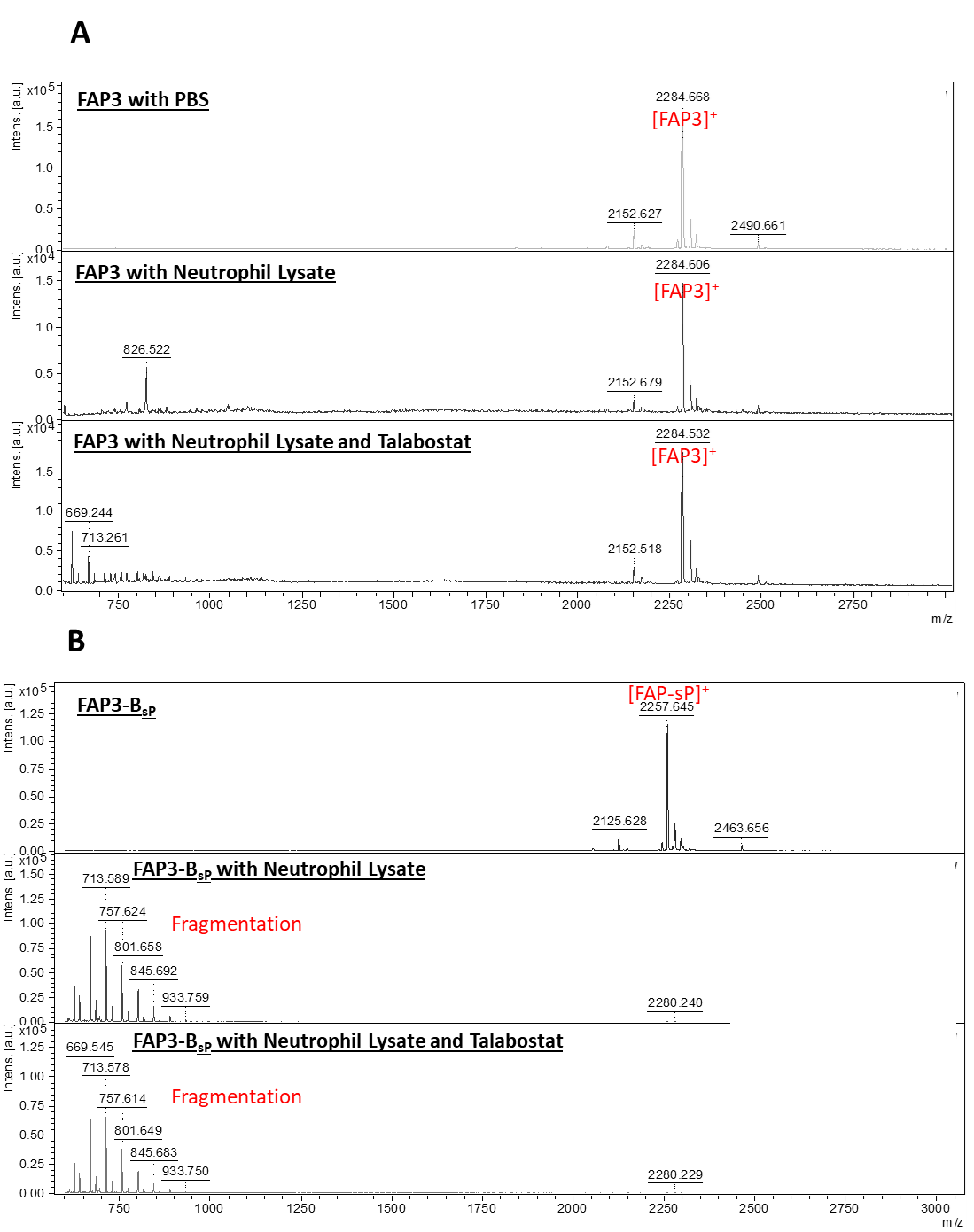


**Figure S3: MALDI analysis showing FAP3 is more stable in neutrophil lysate than FAP-sP. (A)** MALDI analysis confirms FAP3 remains intact when incubated with activated neutrophil lysate; **(B)** MALDI analysis shows FAP-sP is fragmented when incubated with activated neutrophil lysate. Breakdown also occurs in the presence of Talabostat, showing this breakdown is not FAP mediated.


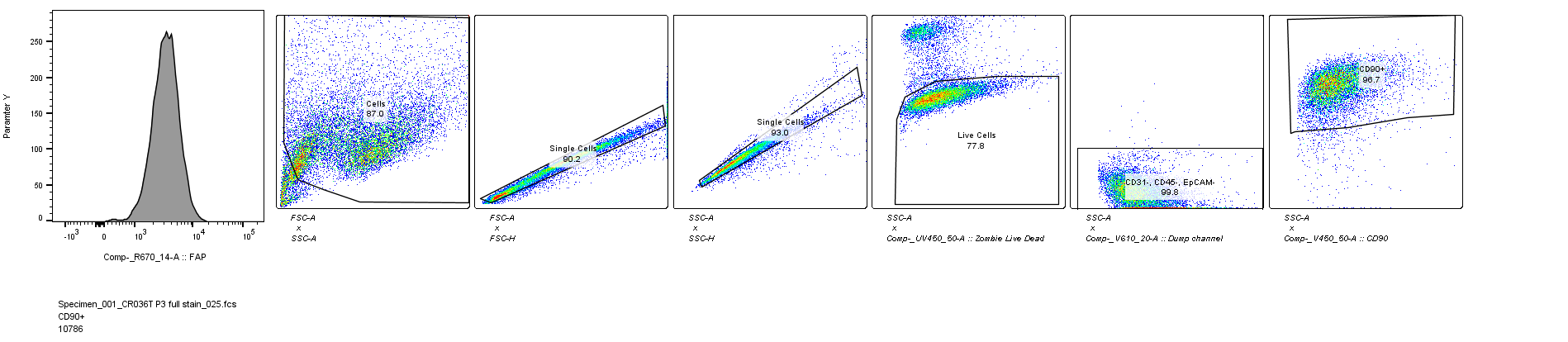

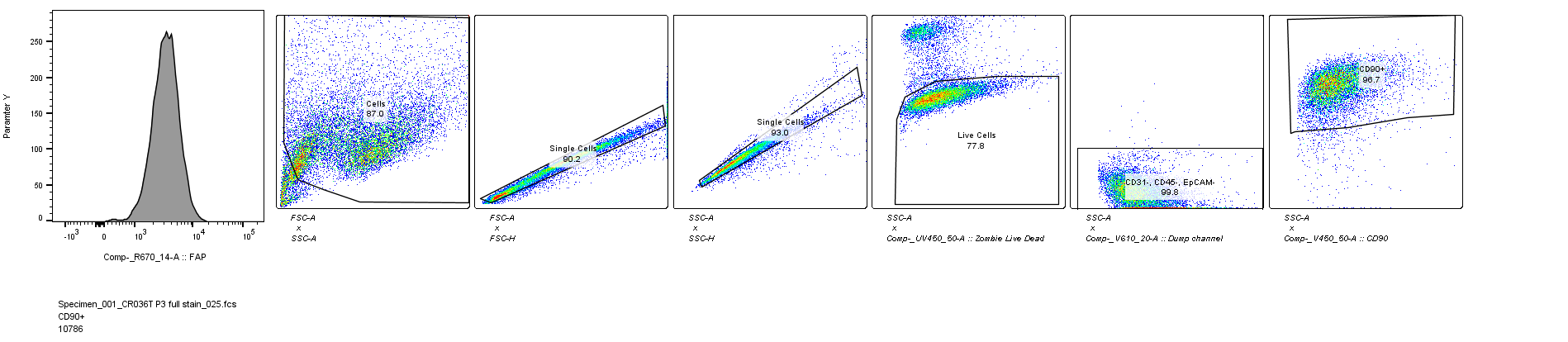


Gate on all cells to exclude debris

Doublets excluded to only consider single cells

Live cells selected by staining with a viability dye

CD31-, CD45-, EpCAM- cells selected to exclude endothelial cells, leukocytes and epithelial cells

CD90+ cells selected - fibroblasts

**Figure S4: Gating strategy used for cultured cancer associated fibroblasts (CAFs).** Gating strategy excludes other cell types to ensure only expression on fibroblasts assessed and then FAP positivity confirmed.


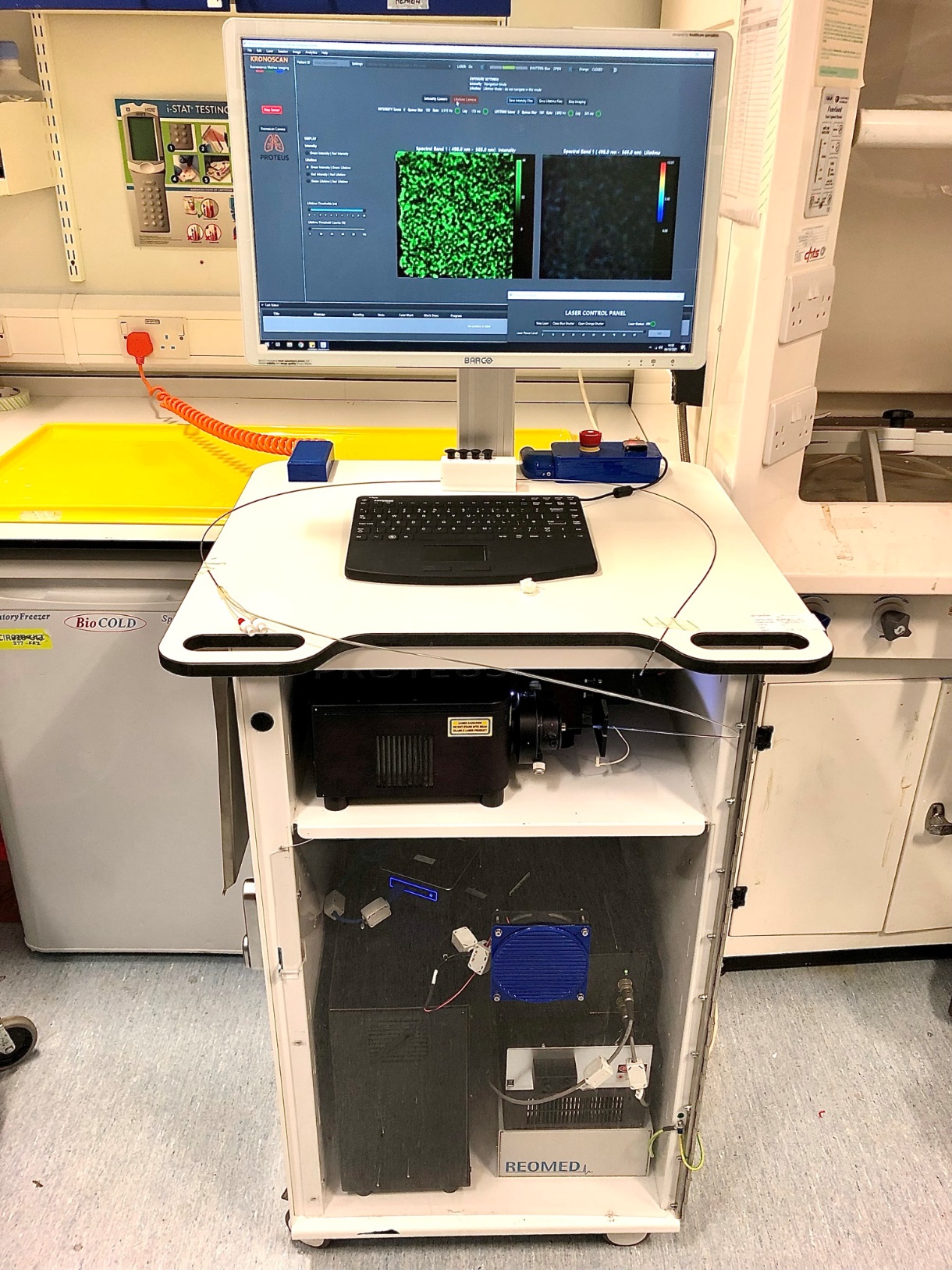
**Figure S5: Clinically approved fibre based fluorescence and lifetime imaging system.** Imaging system shown on a medical cart, which is fibre based and compatible with bronchoscopy procedures.

**Figure S6: Assessment of fluorescence intensity (A) and fluorescence lifetime (B) of FAP3 probe with increasing concentration of recombinant human FAP.** Measurement of fluorescence intensity and lifetime in varying concentrations of rhFAP in solution on a clinically tractable system.

**Figure S7: The lifetime and FLIM measurements of 5 patient samples.** Histological stage and subtype demonstrated on the y-axis and measurements of intensity and lifetime for each sample shown over time, with and without added inhibitor (Talabostat). Dotted line demonstrates the intrinsic values for each sample.
